# Effects of autologous concentrated growth factor on gingival thickness in periodontal accelerated osteogenic orthodontics: a 6-month randomized controlled trial

**DOI:** 10.1186/s12903-021-01967-5

**Published:** 2021-11-23

**Authors:** Lei Qi, Weiwen Ge, Ningning Cao, Shoupeng Wang, Yifeng Qian, Xudong Wang, Lei Zhang

**Affiliations:** 1 Department of Oral and Cranio-Maxillofacial Surgery, Ninth People’s Hospital, Shanghai Jiao Tong University School of Medicine, No. 639 Zhizaoju Road, Shanghai, 200011 China; 2 College of Stomatology, Shanghai Jiao Tong University, National Center for Stomatology, Shanghai, 200011 China; 3 National Clinical Research Center for Oral Diseases, Shanghai Key Laboratory of Stomatology, Shanghai, 200011 China

**Keywords:** Concentrated growth factor, Gingival thickness, Periodontal accelerated osteogenic orthodontics, Randomized controlled trial

## Abstract

**Background:**

Earlier studies have not given clear results of concentrated growth factor (CGF) on gingival thickness (GT) in periodontal accelerated osteogenic orthodontics (PAOO). This randomized controlled trial aimed to evaluate the effects of CGF on GT in patients with thin gingival phenotype undergoing PAOO.

**Methods:**

Forty four patients presenting 264 anterior mandibular teeth were recruited and randomly allocated to one of the groups: test—positioning of autologous CGF after PAOO or control—positioning of a collagen membrane after PAOO. GT, gingival height (GH), buccal alveolar bone thickness (BT), and buccal alveolar bone height (BH) were evaluated depending on cross-sectional CBCT images at *t*0 (before surgery) and *t*1(6 months after surgery).

**Results:**

GT were increased in both groups at *t*1 compared to *t*0. Yet, higher values were observed in the test group (from 0.94 ± 0.23 to 1.31 ± 0.33 mm) compared to the control group (from 0.94 ± 0.19 to 1.02 ± 0.16 mm) (*p* < 0.05). Moreover, in the intergroup comparison, GT at *t*1 in the test group was significantly higher compared to the control group (*p* < 0.01). Furthermore, the GT of central incisors, lateral incisors and canine teeth all showed significantly changes compared with baseline and the test group showed higher increase (*p* < 0.01). No statistically significant difference were found in GH, BT, BH and all clinical parameters between two groups at *t*1 (*p* > 0.05).

**Conclusions:**

Within the limitation of this study, gingival thickness could be increased by using CGF in PAOO for the patients with thin gingival phenotype.

*Trial registration* The study was registered in Chinese Clinical Trial Registry (http://www.chictr.org.cn/index.aspx) under the number ChiCTRINR17013346, Registered 11 November 2017.

**Supplementary Information:**

The online version contains supplementary material available at 10.1186/s12903-021-01967-5.

## Background

Orthognathic and orthodontic joint treatment is widely accepted treatment method for dental and maxillofacial deformities [1–3]. However, compared with regular patients, patients with dentofacial deformities faced many embarrassing problems including dental decompensation, limition of tooth movement and time-consuming in the process of preoperative orthodontic treatment [4, 5]. Especially for Skeletal Class III dentofacial deformities, alveolar bone absorption, roots exposure, and thin gingiva can seriously limit the range of tooth movement and prolong the entire treatment time [6, 7]. Hence, accurate periodontal risk assessment is required to reduce the underlying complications.

So far, several periodontal surgeries have been proposed for solving periodontal tissue problems [8–10]. For example, periodontally accelerated osteogenic orthodontics (PAOO) is a relatively new procedure that has been applied to increase alveolar bone volume, shorten treatment time, increase the scope of treatment, reduce root resorption, and provide greater post-treatment stability [11–15]. However, conventional PAOO using collagen barrier membrane could not increase the gingival thickness (GT). Patients with thin gingival phenotype are more likely to suffer a gingival recession after surgery.

The connective tissue graft (CTG) with a coronally advanced flap (CAF) procedure is the standard treatment approach for increasing gingival thickness [16]. Previous studies have suggested that PAOO could be combined with CTG or acellular dermal matrix to improve GT [17, 18]. he use of CTG in combination with PAOO has some important disadvantages, such as the existence of two surgical sites, increasing post-operative morbidity and limited availability of donor tissue [19]. Moreover, CTG and CAF can not increase the alveolar bone volume of patients with skeletal deformities. Furthermore, the therapy of CTG and PAOO will result in extended surgical time and increased postoperative bleeding and pain [20]. Therefore, alternative methods should be investigated to correct thin gingival phenotype in patients with dentoskeletal deformities treated by PAOO.

Growth factor therapy has recently gained more attention since it has been established; it can improve the effect of periodontal surgery [21]. Concentrated growth factor (CGF) has been used as a promoter for repairing intra-bony defects, fat graft, and sinus augmentation during tissue regeneration [22, 23]. Previous study found that CGF and CAF can improve the gingival recession by increasing the width of keratinized gingiva (KTW) and GT [24]. Our previous study has also demonstrated that CGF promotes gingival regeneration through the AKT/Wnt/β-catenin and YAP signaling pathways [25]. Although tissue regeneration using CGF yields promising clinical and experimental results, so far, no study evaluated the effect CGF in combination with PAOO regarding the GT in patients with skeletal deformities.

The aim of this randomized controlled trial was to evaluate the clinical effectiveness of CGF used in PAOO on GT in patients with skeletal deformities and to compare other clinical parameters with collagen barrier membrane.

## Methods

### Trial design

This study was designed as a randomized controlled clinical trial to the effects of compare collagen barrier membrane (control group) and CGF memberane (test group) in PAOO. Forty-four consecutive patients from the Department of Oral and Cranio-Maxillofacial Surgery, Ninth People’s Hospital, Shanghai Jiao Tong University School of Medicine, who met the inclusion criteria were enrolled from February 2018 to April 2020. The study was registred at Chinese Clinical Trial Registry (ChiCTRINR17013346). The study protocol in this study was approved by the Ethics Committee of Shanghai Ninth People’s Hospital, Shanghai Jiao Tong University School of Medicine (SH9H-018-164-T122). All methods were performed in accordance with the relevant guidelines and regulations approved by the Ethics Committee.

### Sample size

Before the initiation of the study, the power analysis for sample size calculation was performed. In short, the primary parameter is GT. According to the preliminary results, the increase of GT in control group and test group is 0.25 ± 0.11 mm and 0.40 ± 0.21 mm respectively. According to the results of power analysis, a minimum of 22 patients was needed for each group so as to obtain 80% power in our study after considering 10% dropouts.

### Eligibility criteria

The inclusion criteria were the following: (1) male or female patients aged 18–35 years; (2) systemically and periodontally healthy non-smoker patients; (3) cone-beam computed tomography (CBCT) showing gingival thickness (GT) ≤ 1.0 mm; (4) patients with osseous dehiscence; (5) patients with dental or skeletal deformities who needed orthodontic compensation treatment or decompensated orthodontic treatment; (6) signed informed consent. The exclusion criteria were the following: (1) patients with periodontal disease or severe gingival recession; (2) patients with abnormal blood and coagulation functions; (3) patients allergic to implants; (4) use of medicines that may cause gingival enlargement; (5) patients with heart, lung, brain, kidney, and other organs diseases; (6) patients with mental illness; (7) pregnant or lactating females.

### Patient inclusion: informed consent, randomization and allocation concealment

**Fig. 1:**
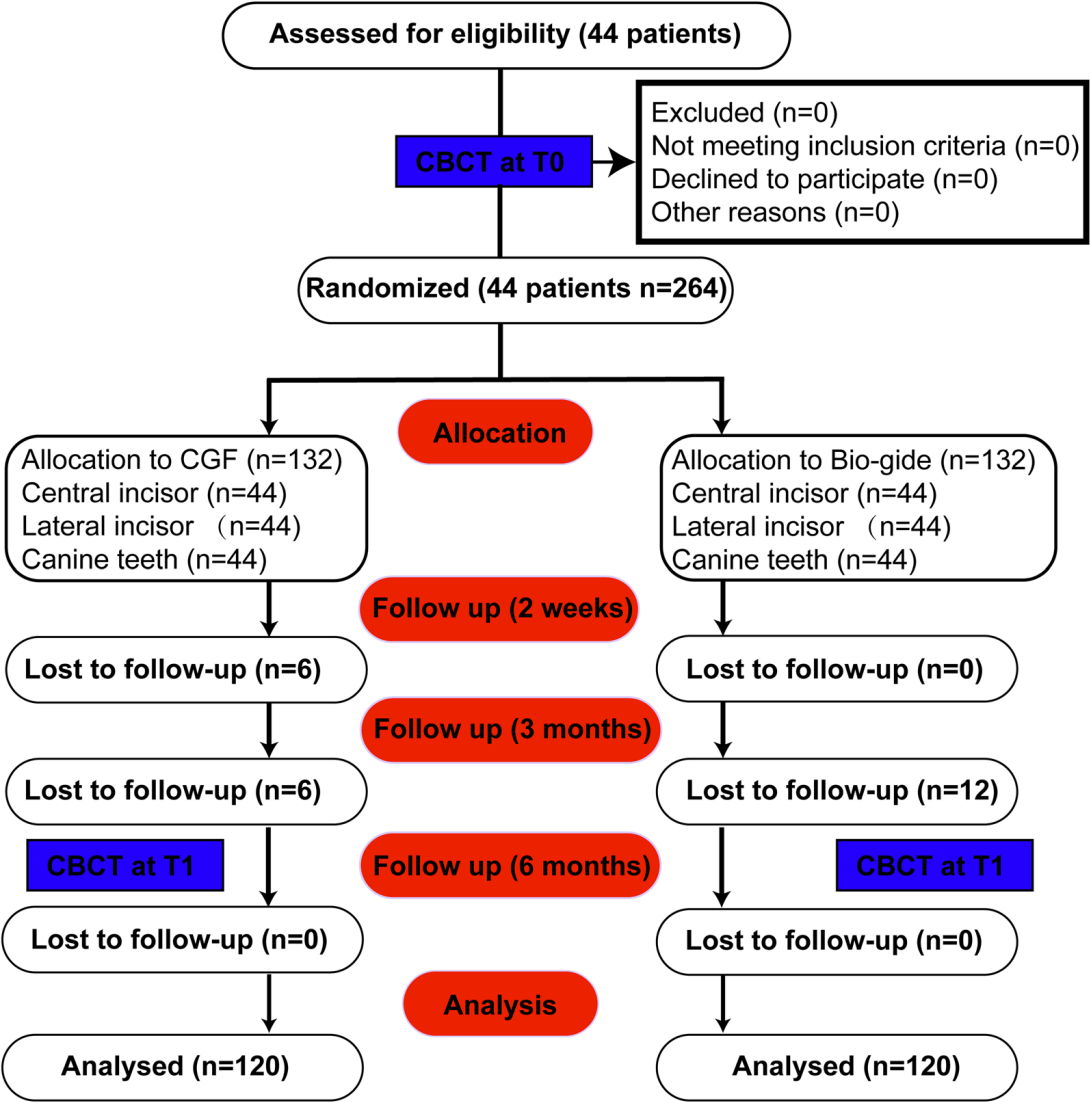
Consort flowchart of the study

After the purposes, risks, benefits, and monitoring of the study were explained, the patients were invited to sign an informed consent form. Patients were randomly assigned to the control group (collagen barrier membrane) or test group (CGF). Sealed envelopes were used to perform randomization with an equal number of envelopes for every group. Group allocation was revealed just before surgery and remained blinded for the evaluating investigator for data collection, analysis, and processing during the project's analytical stage. The location and distribution of subjects are shown in Fig. 1.

### Preoperative preparation

All the patients received full-mouth scaling 2 weeks before surgery. After clinical evaluations including oral hygiene and the condition of periodontal soft and hard tissue, all patients underwent CBCT with the same settings at t0 (before surgery). CBCT images were obtained with the patient using a lip retractor in order to allow visualization of gingival tissues at the images with i-CAT CBCTscanner (Imaging Sciences International, Hatfield, Pa) after setting the acquisition parameters as follows: 120 kV, 5 mA, exposure time 26.9 s, 0.125 mm slice thickness, 8 cm × 8 cm field of view. Then, the evaluating investigator would measure the GT preoperatively to determine whether the patients could participate in the following study.

### Interventions

#### CGF membrane preparation

CGF was prepared from human venous blood obtained from the subjects in test group. All subjects enrolled in this study gave informed consent. Venous blood was collected in two sterile 9 ml tubes and was immediately centrifuged in a special centrifuge (Medifuge, Slifadent srl, Sofifia, Italy) for 13 min. Briefly, this centrifuge device used a program with the characteristics: 2700 rpm 2 min, 2400 rpm 4 min, 2700 rpm 4 min, and 3000 rpm 3 min. As shown in Additional file 1: Fig. S1, the CGF layer was squeezed to obtain CGF membrane using a matched machine.

### Surgical procedure

**Fig. 2:**
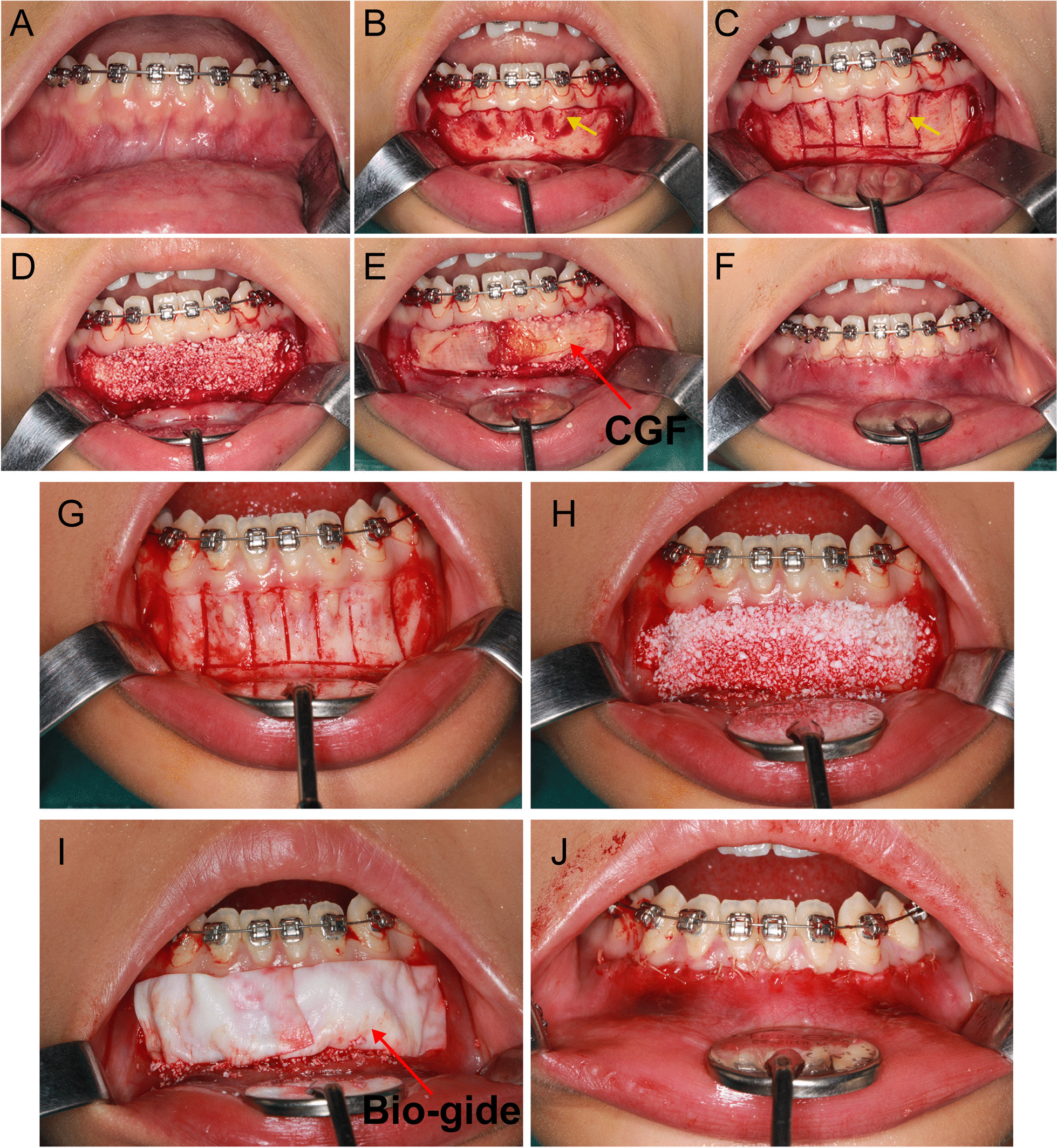
The process of PAOO. **A** The gingival contour of the patient. **B** Flap design. **C**, **G** Decortication. **D**, **H** Bone graft. **E** Covering CGF membrane. **I** Covering Bio-gide membrane. **F**, **J** Close the incision. yellow arrow: osseous dehiscence

As presented in Fig. 2, the surgical technique for PAOO performed in mandibular anterior teeth (33–43) included 5 steps: (1) A full thickness periosteal flap was raised. For esthetic purposes the papilla between every teeth should be preserved on the labial aspects. (2) Vertical grooves were made to extend from a point 2 to 3 mm above the crest of the bone to a point 2 mm beyond the apices of the roots. Horizontal corticotomy cuts were made joining these vertical cuts. (3) Bone graft (0.5 g, Bio-oss, Geistlich Biomaterials, Wolhusen, Switzerland) was implanted in the thin area of the cortical bone. (4) CGF (test group) or collagen barrier membranes (25 mm*25 mm, Bio-gide, Geistlich Biomaterials, Wolhusen, Switzerland; control group)were used to cover bone graft. (5) Flaps were sutured without tension. Sutures were removed after 1 to 2 weeks.

### Postoperative care

After surgery, the oral cavity was kept clean with 0.2% chlorhexidine and oral hygiene instructions were provided at each post-operative visit. Two weeks after the operation, the patient revisited the hospital, and the doctor checked whether there was wound dehiscence, implant infection, and gingival recession; after 3 months, the gingival conditions including gingival mucosa, gingival recession, swelling and pain were evaluated; after 6 months, the CBCT was acquired to evaluate the changes of GT and other clinical parameters by comparing them with the preoperative ones.

### Data collection

#### Gingival thickness (GT)

The GT and its stability over 6 months was the primary outcome of the study. The GT was evaluated using CBCT. Briefly, the sagittal plane of CBCT slide perpendicular to the long axis of the tooth was selected and the long axis of the teeth was identified. The point was then chosen perpendicular to the long axis at the following level: 4 mm apical to the gingival margin. GT was measured from alveolar bone tissue to the outline of the gingival tissues.

#### Gingival margin height (GH), Buccal alveolar bone thickness (BT), and Buccal alveolar bone height (BH)

For each tooth, the GH, BT, and BH were measured at *t*0 and *t*1 perpendicular to the tooth's long axis. GH is measured as from gingival margin to cemento-enamel junction in CBCT images to determine supracrestal marginal tissue height. BT is measured as from the tooth surface to the bone-soft tissue interface (6 mm apical to the cementum-enamel junction). BH is measured as the distance from cementum-enamel junction to the most coronal alveolar crest level in CBCT images.

#### Pain and swelling scores

Pain and swelling were evaluated at 2 weeks, 3 months months, and 6 months after surgery according to the results of questionnaires completed by patients. The pain was evaluated using the following score: 0: no pain; 1: mild pain; 2: moderate pain; 3: severe and continuous pain; 4: severe and continuous pain with changes in blood pressure and pulse. The swelling was evaluated using the following score: 0: no swelling; 1: mild swelling; 2: moderate swelling; 3: severe swelling.

#### Healing index (HI)

The HI, including tissue color, bleeding at palpation, epithelization of incision margins, and presence of Absorbable thread dissolution, was evaluated at 2 weeks, 3 months, and 6 months after surgery. A score of 1 indicated very poor improvement, and 5 indicated an excellent recovery [26, 27].

#### Statistical analysis

In this study, all data were performed using statistical software (version 22.0; SPSS Inc., Chicago, IL, USA). First, the data were tested for normality using the Kolmogorov–Smirnov test. The Mann–Whitney U test was used for the intergroup evaluations after the normality of data failed. When making intragroup comparisons, the Wilcoxon signed-rank test was used to assess changes in the double measurements. The Friedman test was used to assess changes in the triple measurements. Descriptive statistics such as mean ± standard deviation and discrete numeric variables (minimum–maximum) were calculated for the clinical and radiographic parameters. Intra- and inter-assessor reliability was assessed on the basis of five cases that were randomly selected for duplicate registration of GT. A *p* value < 0.05 was considered to be statistically significant.

## Results

### Patient characteristics

Fourty-four patients were recruited in this study. A total of 40 patients completed the study after 4 dropouts. All surgeries were performed uneventfully. No adverse events related to surgicial techniques were recorded in the 6 months follow-up (Additional file 1: Fig. S2). Control group was composed by 10 males and 10 females with a mean age of 26 years. Test group was composed by 11 males and 9 females with a mean age of 25 years in the test group. Both control and test group included mandibular central incisors (n = 20), lateral incisors (n = 20), and canines (n = 20).

### Primary outcome

#### GT

**Fig. 3:**
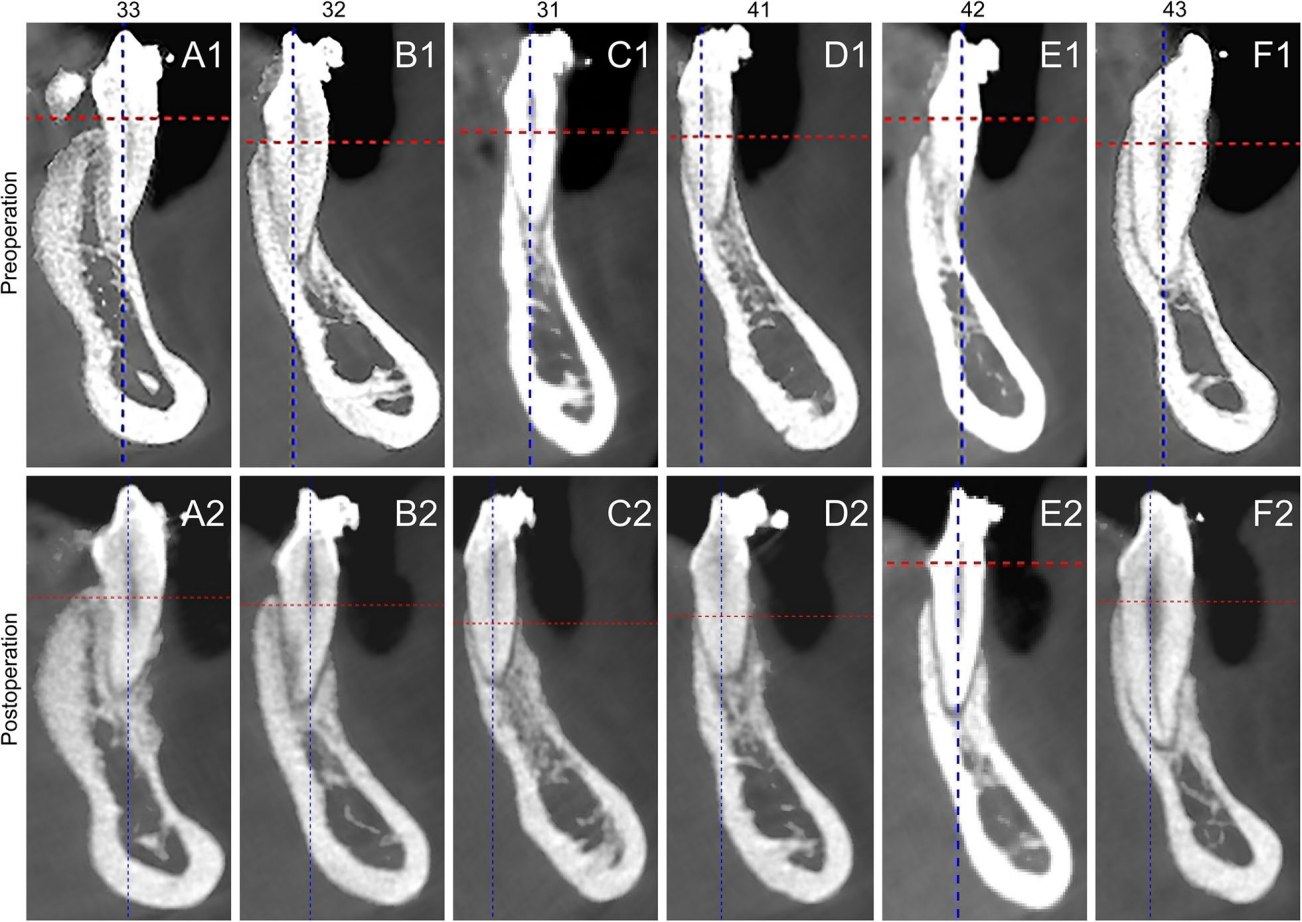
The measurements of CBCT images of central incisors, lateral incisors and canine teeth at *t*0 (preoperation) and *t*1(6 months postoperation) in the test group. 43, 33: canine teeth; 42,32: lateral incisors; 41,31: central incisors

**Fig. 4:**
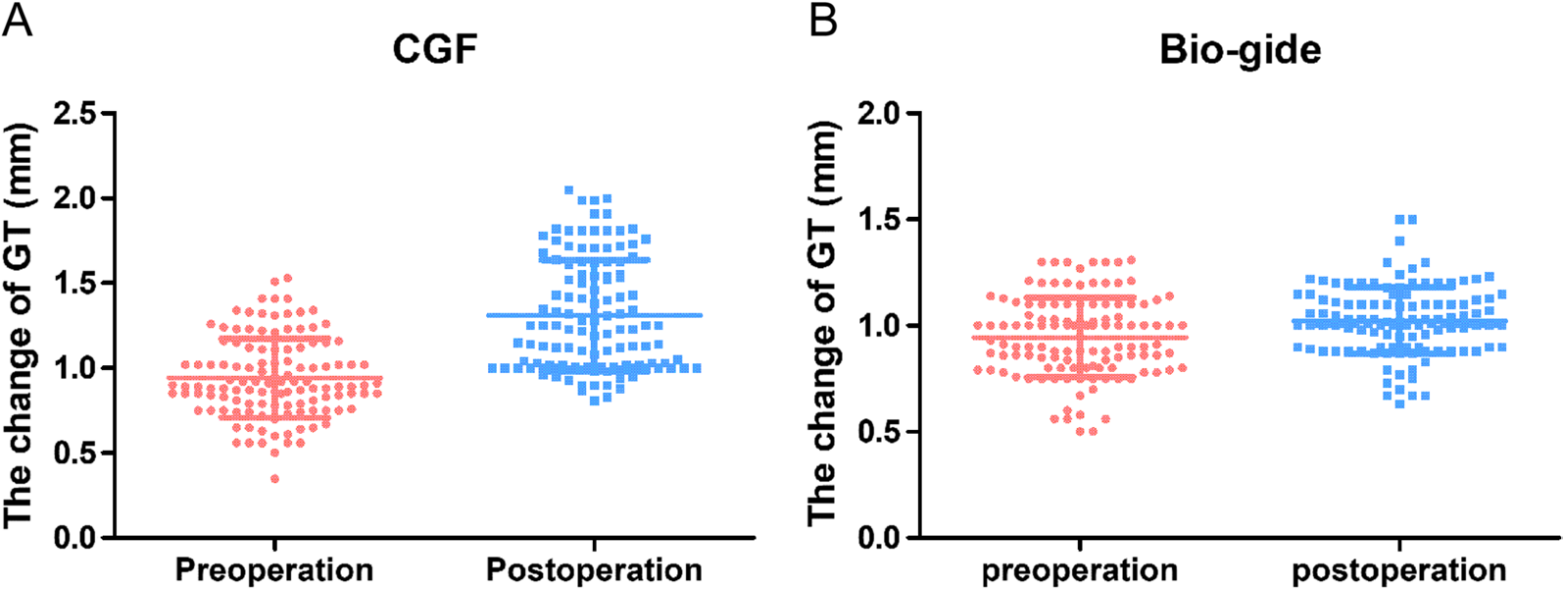
The quantitative assay for the gingival thickness before and 6 months after PAOO using CGF and Bio-gide membrane

The intragroup and intergroup comparisons of radiographic parameters are presented in Fig. 3, Table 1 and Additional file 1: Fig. S3. No significant differences in GT were found between the two groups at *t*0 (*p* = 0.733). As shown in Fig. 4, at *t*1, the mean GT values in the test group were higher compared to the control group (in the test group, the values increased from 0.94 ± 0.23 to 1.31 ± 0.33 mm; in the control group, the values increased from 0.94 ± 0.19 to 1.02 ± 0.16 mm; *p* < 0.01). In the intergroup comparison at *t*1, GT in the test group was significantly higher compared to the control group (*p* < 0.01).

**Table 1:** Descriptive statistics of the clinical parameters measured at baseline and 6 months after surgery

	Control group	Test group	*p*^a^
GT			
Baseline	0.94 ± 0.19 (0.50–1.31)	0.94 ± 0.23 (0.35–1.53)	0.733
6 months	1.02 ± 0.16 (0.63–1.50)	1.31 ± 0.33 (0.81–2.05)	0.000**
*p*^b^	0.000**	0.000**	
GH			
Baseline	2.61 ± 0.51 (2.01–3.80)	2.60 ± 0.49 (1.76–3.90)	0.957
6 months	2.69 ± 0.42 (2.01–4.11)	2.67 ± 0.35 (2.00–3.39)	0.761
*p*^b^	0.064	0.445	
BT			
Baseline	1.04 ± 0.48 (0.91–1.14)	1.04 ± 0.04 (0.94–1.15)	0.568
6 months	1.43 ± 0.04 (1.31–1.56)	1.43 ± 0.06 (1.32–1.58)	0.620
*p*^b^	0.000**	0.000**	
BH			
Baseline	2.67 ± 0.69 (1.89–3.99)	2.71 ± 0.66 (1.21–3.85)	0.055
6 months	1.71 ± 0.44 (1.01–2.18)	1.70 ± 0.48 (1.01–2.54)	0.088
*p*^b^	0.000**	0.000**	

Data are expressed as the mean ± standard deviation (minimum–maximum)

GT, gingival thickness; GH, gingival height; BT, buccal alveolar bone thickness; BH, buccal alveolar bone height;

^a^Mann Whitney U Test, statistically different between groups (**p < 0.01, *p < 0.05)

^b^Wilcoxon Signed Rank Test, significantly different compared with baseline (**p < 0.01,*p < 0.05)

To further observe every surgical site's change, the GT values of central incisors, lateral incisors, and canine teeth were evaluated (Table 2). No significant differences in GT between groups were found at t0. In the control group, the mean GT values of central incisors were increased from 0.94 ± 0.18 to 1.06 ± 0.16 mm at t1 (*p* = 0.000). In the lateral incisors, GT increased from 0.92 ± 0.20 to 0.99 ± 0.17 mm at *t*1 (*p* = 0.009), while in canines there was an increase from 0.98 ± 0.18 to 1.02 ± 0.13 mm (p = 0.129). In the test group, central and lateral incisors and canines showed a significant increase in GT from t0 to t1 (p < 0.05). GT was significantly greater in central and lateral incisiors and canines of test than control group at t1.

**Table 2:** Descriptive statistics of gingival thickness of central incisors, lateral incisors and canine teeth measured at baseline and 6 months after surgery

	Control group	Test group	*p*^a^
Central incisors			
Baseline	0.94 ± 0.18 (0.50–1.27)	0.94 ± 0.28 (0.35–1.53)	0.783
6 months	1.06 ± 0.16 (0.73–1.50)	1.31 ± 0.34 (0.81–1.99)	0.009**
*p*^b^	0.000**	0.000**	
Lateral incisors			
Baseline	0.92 ± 0.20 (0.56–1.31)	0.96 ± 0.23 (0.50–1.51)	0.479
6 months	0.99 ± 0.17 (0.67–1.50)	1.30 ± 0.33 (0.94–2.05)	0.000**
*p*^b^	0.009**	0.000**	
Canine teeth			
Baseline	0.98 ± 0.18 (0.56–1.30)	0.94 ± 0.20 (0.60–1.34)	0.262
6 months	1.02 ± 0.13 (0.63–1.30)	1.32 ± 0.32 (0.90–2.00)	0.000**
*p*^b^	0.129	0.000**	

Data are expressed as the mean ± standard deviation (minimum–maximum)

^a^Mann Whitney U Test, statistically different between groups (**p < 0.01, *p < 0.05)

^b^Wilcoxon Signed Rank Test, significantly different compared with baseline (**p < 0.01,*p < 0.05)

### Secondary outcome

#### GH

There was no statistically significant difference between control and test group in the intergroup comparison at *t*0 (*p* = 0.957; Table 1) and *t*1 (*p* = 0.761). GH values were increased from 2.61 ± 0.51 to 2.69 ± 0.42 mm (*p* = 0.064) in the control group. And in the test group, GH values were increased from 2.60 ± 0.49 to 2.67 ± 0.35 mm (*p* = 0.445).

#### BT

No significant differences between groups were observed t0 and t1 (p > 0.05; Table 1). After 6 months, BT increased from 1.04 ± 0.48 to 1.43 ± 0.04 mm in the control group (*p* < 0.01) and from 1.04 ± 0.04 to 1.43 ± 0.06 mm in the test group (*p* < 0.01).

#### BH

No significant differences were found between groups at t0 and t1 (p > 0.05; Table 1). After 6 months, BH increased from 2.67 ± 0.69 to 1.71 ± 0.44 in the control group (*p* < 0.01) and from 2.71 ± 0.66 to 1.70 ± 0.48 in the test group (*p* < 0.01).

### Clinical outcomes

No significant differences in pain scores were found between groups at 3 and 6 months after surgery (*p* > 0.05; Additional file 1: Table S1). However, at 2-week observation period, a significantly lower score was found in test compared to control group (*p* = 0.024).

There was no statistically significant difference in mean swelling and HI values between groups at 2 weeks, 3 months, and 6 months. A significant intragroup difference in pain and swelling scores were observed for both groups at 2 weeks (*p* < 0.01). Moreover, mean HI values were significantly increased in both groups at 3 months and 6 months when compared with the second week (*p* < 0.01).

## Discussion

This study was designed as a randomized and controlled clinical trial of patients with skeletal deformities. Our data suggested that CGF could increase the GT of mandibular anterior teeth after PAOO to some degree, which is consistent with our previous basic research results. It has to be pointed out that skeletal deformities may increase surgical difficulties compared with normal patients, as the surgical field has more anatomic variations. Moreover, thin gingival phenotype is common in patients with skeletal deformities, resulting in a higher risk of developing gingival recession after PAOO. Considering that, the use of CGF membrane could favor bone regeneration and soft tissue augmentation at the same time.

Poor gingival condition was one of common problems of skeletal deformities. Anatomical factors, malocclusion, and dental compensation are the main causes of poor gingival conditions [28]. Hence, gingival risk assessment and multidisciplinary treatment are of great importance for clinicians when selecting best treatment techniques to avoid treatment failure. The implementation of PAOO could increase the alveolar bone volume, accelerate the movement of orthodontic teeth, and increase pre-orthodontic stability [29]. Although the collagen barrier membranes used in PAOO have many benefits, including soft tissue in-growth exclusion in the defect region and stabilization of the grafting material, they have no clear positive effect on the increase of GT [30, 31]. In addition, gingival recession and root exposure often occur in patients with thin gingival phenotype after PAOO. Hence, increasing GT and improving gingival stability in patients with skeletal deformities are important preoperative orthonathic treatment issues.

Autogenous soft tissue grafts is considered the gold standard treatment approach for improving GT. However, this approach has various drawbacks, such as donor-site damage, limited palatal mucosal tissue, and poor post-operative outcome [32]. Therefore, modified PAOO techniques could increase the GT while increasing the amount of alveolar bone volume needs further exploration. Autologous platelet concentrates containing different growth factors have an essential role in tissue regeneration [33]. CGF, which can be found in a liquid state or as gel or membrane, could adapt to different clinical needs. CGF can enhance bone regeneration over a longer period by promoting cell proliferation, migration, and vascularization [34, 35]. Nevertheless, the underlying mechanisms of CGF-mediated gingival regeneration remain unclear. In our previous study, we found that CGF may directly promote the proliferation and migration of GMSCs in vitro and enhance microcirculation in vivo. Based on these results, in the present study, we further examined the effect of CGF on gingival thickness and other radiographic and clinical results after PAOO.

Our data indicated that either CGF or collagen barrier membrane yielded short-term stable results. Nevertheless, the increase in GT was significantly higher in the test group than the control group after 6 months, which is consistent with a previous study that reported an increase of 0.06 ± 0.34 mm for the CAF group 0.32 ± 0.10 mm for the CAF and CGF group (*p* = 0.000) [24]. Aroca et al. also showed a significant increase in GT in the MCAF + PRF group [36]. The higher increase in GT in the test group may be because CGF contains much larger, denser, and richer growth factors and fibrin matrix [37]. Taken together, these results confirmed that CGF could effectively increase GT. To the best of our knowledge, this is the first study that investigated the effect of CGF on GT of different surgical sites. As presented in Table 2, the GT values of all mandibular anterior teeth in the test group at *t*1 were significantly different compared to *t*0. In the control group, the mean GT values of the central incisors and canines at *t*1 were statistically significant compared with *t*0, while the lateral incisors did not show a significant change in terms of the mean GT values. This may be related to the complex anatomy and surgical sites [38].

The other major finding in the present study was the increase in gingival height (GH) in the test group. GH is an important factor for the gingival function and aesthetic, responsible for reducing the incidence of periodontal disease and the maintenance of periodontal health. A previous study suggested that CGF could increase KTW by 0.58 mm, which was beneficial in terms of attachment gain and increased long-term stability [24]. In this study, the mean GH in the test group showed an increase of 0.04 mm at *t*1 compared to an increase of 0.01 mm in the control group. The differences from the previous study may lay in different surgical and study designs as we only included the anterior mandibular teeth. Muscles in the maxilla and mandible have different tensile strength, and the differences in flap thicknesses may produce different results. Also, molar teeth have a larger surgical field and more complex anatomy, which was not considered in the present study. Hence, whether CGF could affect the increase of GH remains to be explored. However, no gingival recession during the PAOO occurred regardless of using CGF or collagen barrier membrane.

There was a significant increase in BT and BH from t0 to t1 in both groups, but no differences in these parameters were found between groups. The increase in bone volume in both groups is expected, since both groups were bone grafted, which contributed to a decrease in recession depth as well. Previous studies have reported that CGF has a positive effect on bone regeneration and wound healing, as it stimulates the fibroblast-DNA synthesis and promotes the expression of chemotactic factors, thus attracting osteoblastic cells to the wound area [39]. This point could not be reflected in our present study due to the influence of bone augmentation during PAOO, which needed further histological examination.

CGF can accelerate soft tissue healing because of its growth and fibrin network structure. Several studies have indicated that PRF could decrease post-operative pain, increase the mean HI values, and accelerate healing during the first weeks [40, 41]. In this study, a significant difference in the mean pain values was observed between the control and test groups at two weeks after surgery. However, there was no significant difference in mean swelling and HI values between control and test groups when performing the intergroup comparison. PAOO was a relatively large periodontal surgery that involved at least six teeth surgical sites. Avoiding excessive stripping of mucoperiosteum and reducing operation time may be essential to decrease the pain, swelling, and HI values. Therefore, post-operative discomfort, including pain and swelling after surgery, is common, especially in the first three days. In this study, no significant difference in terms of swelling and HI was observed between the test and control group at 2 weeks after surgery.

However, when interpreting the present study results, the following limitations should be taken into account. First, the 6-month post-operative measurement period was chosen to evaluate the gingival and alveolar bone indicators' stability after PAOO. The long-term evaluation is of utmost importance and may have a relevant impact on clinical decision-making and promoting the application of this novel strategy. Second, this study only included mandibular anterior teeth. The effects of CGF on posterior and maxillary teeth need to be investigated by further clinical studies because of the anatomic variations of surgical fields that may affect the results. Third, although CBCT images are characterized by high spatial resolution, soft tissues' visibility has limitations. Hence, according to these outcome variables, further RCTs should be designed to verify reported findings and establish an optimal treatment approach.

## Conclusion

Within the limits of the study, it can be concluded that CGF could increase gingival thickness in patients with skeletal deformities who underwent PAOO. PAOO combined with CGF also seems to be an effective option for BT and BH improvement and alveolar bone reestablishment.

## Supplementary Information

**Additional file 1: Fig. S1.** CGF membrane preparation. A: The CGF fibrin block was squeezed with a apecial box. B: CGF membrane and its length. **Fig. S2**. The images of follow-up. A, D: 2 weeks post-operative images; B, E: 3 months post-operative images; C, F: 6 months post-operative images. **Fig. S3**. The measurements of CBCT images of central incisors, lateral incisors and canine teeth at t0 ( before durgery) and t1( 6 months after surgery) in the control group. 43, 33: canine teeth; 42,32: lateral incisors; 41,31: central incisors. **Table S1**. Postoperative pain, swelling and healing evaluations.

## Data Availability

The datasets used and/or analysed during the current study available from the corresponding author on reasonable request.

## Abbreviations

CGF: Concentrated growth factor
GT: Gingival thickness
PAOO: Periodontal accelerated osteogenic orthodontics
GH: Gingival height
BT: Buccal alveolar bone thickness
BH: Buccal alveolar bone height
CTG: Connective tissue graft
CAF: Coronally advanced flap
KTW: Keratinized gingiva
CBCT: Cone-beam computed tomography
