# Supplementary material for: Effects of autologous concentrated growth factor on gingival thickness in periodontal accelerated osteogenic orthodontics: a 6-month randomized controlled trial
Source: BMC Oral Health. 2021 Nov 23;21:604. doi: 10.1186/s12903-021-01967-5 (PMC8609726; doi:10.1186/s12903-021-01967-5)
Supplement: Supplementary file 1 — Additional file 1: Fig. S1. CGF membrane preparation. A: The CGF fibrin block was squeezed with a apecial box. B: CGF membrane and its length. Fig. S2. The images of follow-up. A, D: 2 weeks post-operative images; B, E: 3 months post-operative images; C, F: 6 months post-operative images. Fig. S3. The measurements of CBCT images of central incisors, lateral incisors and canine teeth at t0 ( before durgery) and t1( 6 months after surgery) in the control group. 43, 33: canine teeth; 42,32: lateral incisors; 41,31: central incisors. Table S1. Postoperative pain, swelling and healing evaluations. [file 12903_2021_1967_MOESM1_ESM.pdf]

Title: Effects of Concentrated Growth Factor on Gingival Thickness in Periodontal Accelerated Osteogenic Orthodontics: A 6-month Randomized Controlled Trial

Authors: Lei Qi*, Weiwen Ge*, Ningning Cao, Shoupeng Wang, Yifeng Qian, Xudong Wang, Lei Zhang

Supplementary Figure 1 CGF membrane preparation.

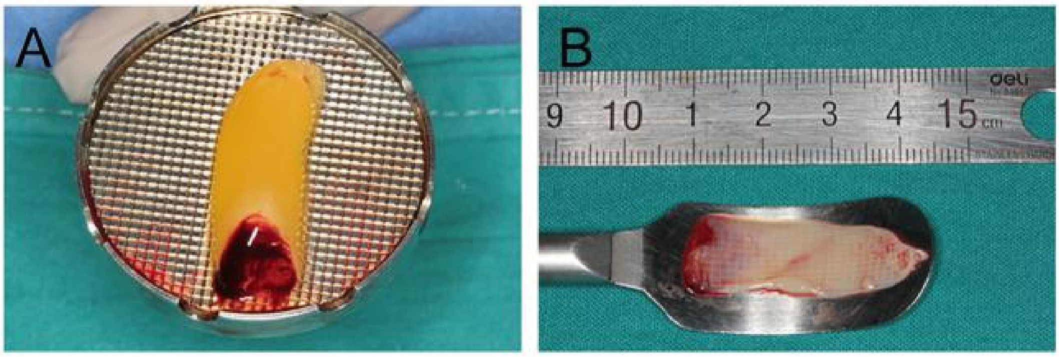

A: The CGF fibrin block was squeezed with a apecial box. B: CGF membrane and its length.

Supplementary Figure 2 The process of PAOO in control group.

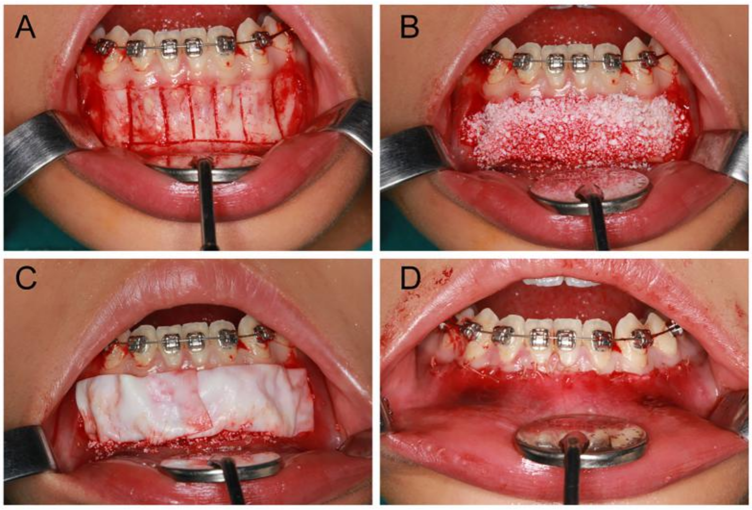

A: Flap design. B: Decortication and bone graft. C: Covering Bio-gide membrane. D: Close the incision.

Supplementary Figure 3

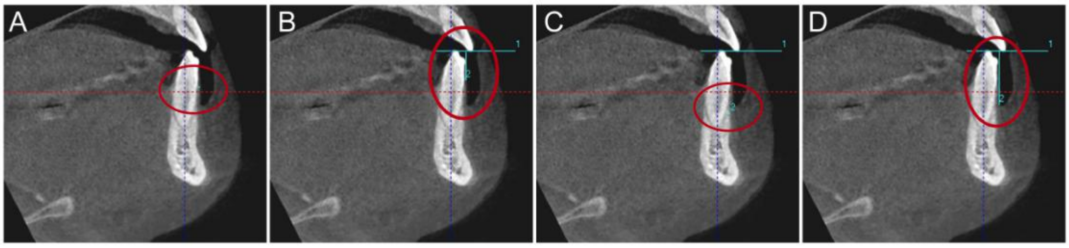

Illustration showing how the gingival thickness (GT), gingival margin height (GH), buccal alveolar bone thickness (BT), and buccal alveolar bone height (BH) were measured on CBCT images. Red circle: the region of measurement.

Supplementary Figure 4 The images of follow-up.

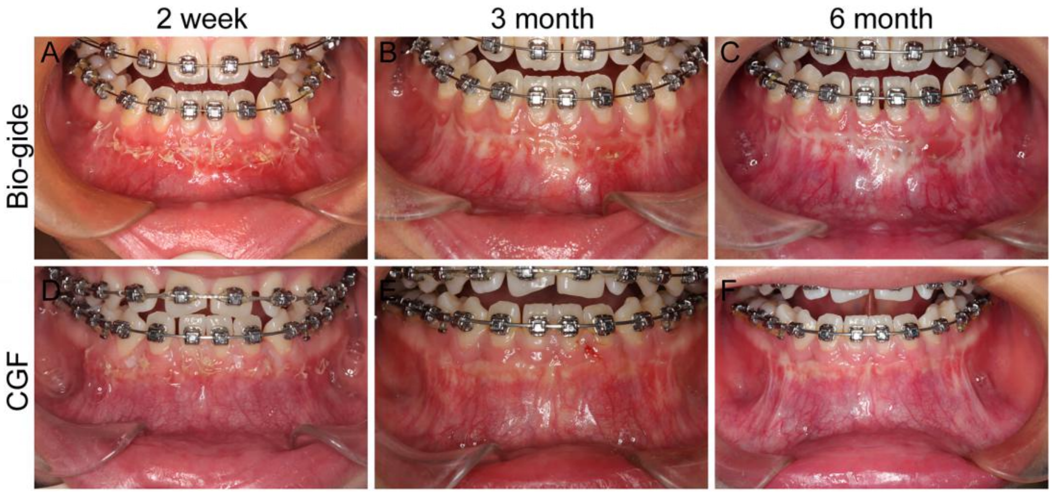

A, D: 2 weeks post-operative images; B, E: 3 months post-operative images; C, F: 6 months post-operative images.

Supplementary Figure 5

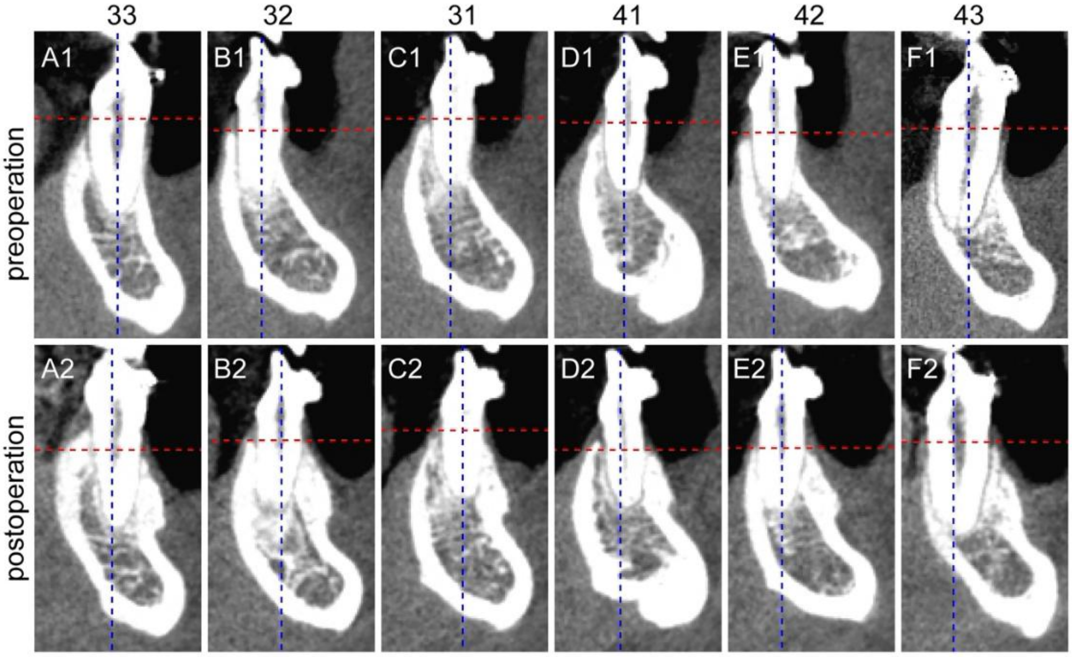

The measurements of CBCT images of central incisors, lateral incisors and canine teeth at *t*0 (before durgery) and *t*1(6 months after surgery) in the control group. 43, 33: canine teeth; 42,32: lateral incisors; 41,31: central incisors.

Supplementary Table 1 Postoperative pain, swelling and healing evaluations

	Control group	Test group	p^a
Pain			
2 weeks	2.15±0.67（1.00-3.00）	1.80±0.52（1.00-3.00）	0.024*
3 months	0.00±0.00（0.00-0.00）	0.00±0.00（0.00-0.00）	1.000
6 months	0.00±0.00（0.00-0.00）	0.00±0.00（0.00-0.00）	1.000
p^b	0.000**	0.000**	
Swelling			
2 weeks	1.60±0.68（1.00-3.00）	1.55±0.69（1.00-3.00）	0.786
3 months	0.00±0.00（0.00-0.00）	0.00±0.00（0.00-0.00）	1.000
6 months	0.00±0.00（0.00-0.00）	0.00±0.00（0.00-0.00）	1.000
p^b	0.000**	0.000**	
HI			
2 weeks	3.00±0.73（2.00-4.00）	2.95±0.69（2.00-4.00）	0.824
3 months	4.55±0.51（4.00-5.00）	4.65±0.49（4.00-5.00）	0.524
6 months	5.00±0.00（5.00-5.00）	5.00±0.00（5.00-5.00）	1.000
p^b	0.000**	0.000**	

Data are expressed as the mean±standard deviation (minimum-maximum)

HI, healing index;

a Mann Whitney U Test, statistically different between groups (**$p<0.01$, *$p<0.05$).

b Friedman Test, significantly different compared with baseline (**$p<0.01$,*$p<0.05$).
